# Robust in-silico identification of Cancer Cell Lines based on RNA and targeted DNA sequencing data

**DOI:** 10.1038/s41598-018-36300-8

**Published:** 2019-01-23

**Authors:** Raik Otto, Jan-Niklas Rössler, Christine Sers, Soulafa Mamlouk, Ulf Leser

**Affiliations:** 1 Knowledge Management in Bioinformatics, Institute for Computer Science, Humboldt-Universität zu Berlin, Unter den Linden 6, 10099 Berlin, Germany; 2 Charité Universitätsmedizin Berlin, Institute of Pathology, 10117 Berlin, Germany; 3 German Consortium for Translational Cancer Research, Berlin, Germany

## Abstract

Cancer cell lines (CCL) are an integral part of modern cancer research but are susceptible to misidentification. The increasing popularity of sequencing technologies motivates the in-silico identification of CCLs based on their mutational fingerprint, but care must be taken when identifying heterogeneous data. We recently developed the proof-of-concept Uniquorn 1 method which could reliably identify heterogeneous sequencing data from selected sequencing technologies. Here we present Uniquorn 2, a generic and robust in-silico identification method for CCLs with DNA/RNA-seq and panel-seq information. We benchmarked Uniquorn 2 by cross-identifying 1612 RNA and 3596 panel-sized NGS profiles derived from 1516 CCLs, five repositories, four technologies and three major cancer panel-designs. Our method achieves an accuracy of 96% for RNA-seq and 95% for mixed DNA-seq and RNA-seq identification. Even for a panel of only 94 cancer-related genes, accuracy remains at 82% but decreases when using smaller panels. Uniquorn 2 is freely available as R-Bioconductor-package ‘Uniquorn’.

## Introduction

Cancer Cell Lines (CCLs) are a critical tool for cancer researchers which facilitate the reproduction of biological experiments, help investigate cancer etiology and aid in the functional characterization and validation of driver mutations. Additionally, usage of CCLs avoids ethical and legal issues when compared to patient-based studies^1–4^. CCLs are, however, susceptible to misidentification and cross-contamination^1,5–8^. A well-known case of misidentification that negatively affected a wide range of researchers was the confusion of the widely used MDA-MB-435 mammary CCL with the M14 melanoma CCL^9^. No nomenclature system that could help avoid idiosyncratic and misleading CCL-names has been universally adopted so far, leading to highly bewildering naming ambiguities such as ‘TT’ (CCL derived from thyroidal tissue) and ‘T.T’ (CCL derived from esophageal tissue), which are different CCLs with almost identical names^10^. Another example that underlines that CCL names cannot be reliably utilized to infer their relationship are the NCI/ADR-RES derived from the OVCAR-8; two CCLs with a common origin but significantly different names, obscuring their close relationship^1,8,11^. In total, 15–20% of all CCLs are misidentified^1,12^, while 18–36% are cross-contaminated^13,14^. Accordingly, many journals currently require authors to ensure identity of the CCLs they employed in experiments upon publication. There is, therefore, an underlining and pressing need for identification methods able to detect these critical sources of erroneous data in CCLs.

**Figure 1:**
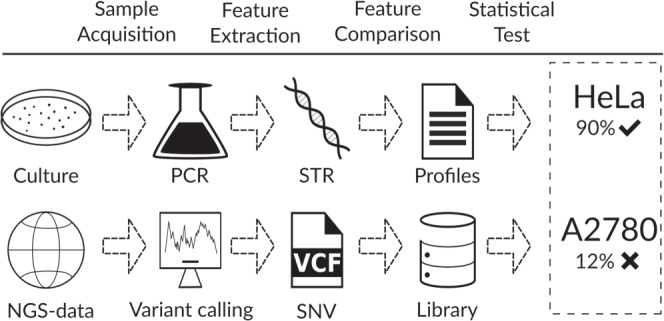
Comparison of the gold-standard in-silico identification methods with Uniquorn 2. The gold-standard ‘short tandem repeat counting’ (STR) method (top) compares tandem counts at specific genomic loci. STR-counts are generally unavailable in NGS-data and therefore, a CCL whose NGS data is available has to be additionally STR-genotyped which requires the physical availability of the to-be-identified CCL sample to conduct a polymerase chain reaction (PCR). Even in-silico identification methods that can utilize NGS-derived Single-Nucleotide Polymorphisms (SNPs) are dependent on the genotyping of the loci that harbor the SNPs. SNP-calls of specific loci however, may not be available due to panel sequencing of the to-be-identified CCL or are incomparable due to utilization of divergent sequencing platforms and filtering of SNP during driver-mutation identification. The Uniquorn 2 in-silico workflow (bottom) requires neither physical availability nor genotyping of specific loci but in contrast works with every NGS-technology that genotypes small variants. Uniquorn 2 does require sets of reference CCLs, called libraries, to match the variants of the to-be-identified CCL and the reference CCLs. After calculating the variant overlap, a statistical test determines whether a variant overlap is sufficiently unlikely to occur by chance in which case the unknown CCL is predicted to be identical to the reference CCL i.e. is identified.

Traditionally, such identification is carried out using specific assays such as Short-Tandem Repeat (STR) genotyping^15^, SNP panel identification assay (SPIA)^5^, MinION^16^ or Multiplex Cell Authentication (MCA)^17^. These assays are costly to perform, time consuming and require physical availability of all samples^18^. An increasingly attractive alternative or complement to such experiments is the in-silico identification of CCLs based on features of their DNA or RNA sequence^5,16,17^. In this setting, only the sequence information of the to-be-identified CCL (termed query) and CCLs of a reference-collection (termed reference library) are used. This has several advantages: sequence features of the CCLs in the reference library can be obtained once and distributed electronically (no physical access required). Additionally, sequence features of the query CCL are often by-products of the original experimentation (no additional cost). The comparison of the features can be performed quickly and in-silico without additional experimental efforts. Figure 1 compares the in-silico with the *in-vitro* approach. However, in practice such an approach can be difficult, as sequencing scope, method and the processing technology used to obtain the features of the reference library are often different from those of the query CCL, leading to notable differences in the resulting sequence features. In a previous work^18^ we presented Uniquorn 1, a robust algorithm for in-silico CCL identification. However, Uniquorn 1’s statistical model was specifically designed for comparing features derived from whole exome sequences. It cannot be applied if, for instance, the reference CCL were exome sequenced, but only the transcriptome or only a panel of genes of the query CCL is available.

In this paper, we present Uniquorn 2, a robust in-silico CCL-identification method that can cope with a much larger heterogeneity between the sequence profiles to be compared than the first version of Uniquorn. In particular, it can compare DNA-derived features with those derived from RNA sequencing, and its model is robust enough to compare sequences of largely different scopes, such as exome sequences with those derived from a gene panel (Table 1).

**Table 1:** Differences between and commonalities of Uniquorn 1 and Uniquorn 2. Uniquorn 2 significantly extends Uniquorn 1 with respect to covered samples sizes, NGS-technologies and data processing. Furthermore, Uniquorn 2 is benchmarked on a much wider and much more heterogeneous set of CCLs. *SNP-filtering refers to the post-sequencing of sequencing data regarding SNPs, such as filtering based on minor allele frequencies.

Property	Uniquorn 1	Uniquorn 2
Technologies covered	DNA	DNA RNA
# of samples used for benchmarking	1984	3896
# of variants used within benchmarks	0.97 million	151 million
Benchmarked comparisons	Hybrid-capture, Exome-seq	Panels, Hybrid-capture, Exome-seq, Full-transcriptome
SNP-filtering*	Yes	No (RNA-seq, panel-seq)

We benchmarked Uniquorn 2 by identifying all identity-relationships in a set of 1612 RNA-sequenced CCLs (5309 related) and in a mixed set of 3596 RNA and DNA-sequencing CCL-profiles (11512 related). Ninety-six% of the relationships of the later RNA-seq CCL-profiles were correctly identified and 95% of the relationships were found in the mixed scenario i.e. when DNA-seq samples were used to identify RNA-seq samples and vice versa. A panel-seq scenario was benchmarked by synthetically limiting the 3596 mixed-scenario samples to the set of genes contained in the Clearseq/Agilent, TruSight/Illumina and Hotspot v2/Thermo Fisher panel, respectively. Panel-sequencing showed sensitivities of 83% (151 genes, Clearseq), 82% (94 genes, TruSight) and 65% (49 genes, Hotspot v2). The algorithm is freely available as R package ‘Uniquorn’ and contains the NCI-60 CCLs by default. Scientists can identify their own custom CCL-samples as well as publicly available CCL-samples.

## Results

### Identification of sequenced CCLs

CCLs are essential tools for cancer research but are also highly susceptible to misidentification, which makes the accurate identification of a CCL used in an experiment crucial. We recently published Uniquorn 1, a method to identify CCLs using variant profiles derived from exome DNA-sequencing or from hybrid-capture DNA-sequencing. Here we present Uniquorn 2 which can robustly identify RNA and panel-sequenced CCLs derived from heterogeneous sequencing technologies while retaining Uniquorn 1’s ability and performance to identify DNA-sequenced CCLs. Furthermore, Uniquorn 2 no longer relies on SNP-filtering, which brings its own problems (such as the concrete set of SNPs to filter) when using pre-computed profiles.

**Figure 2:**
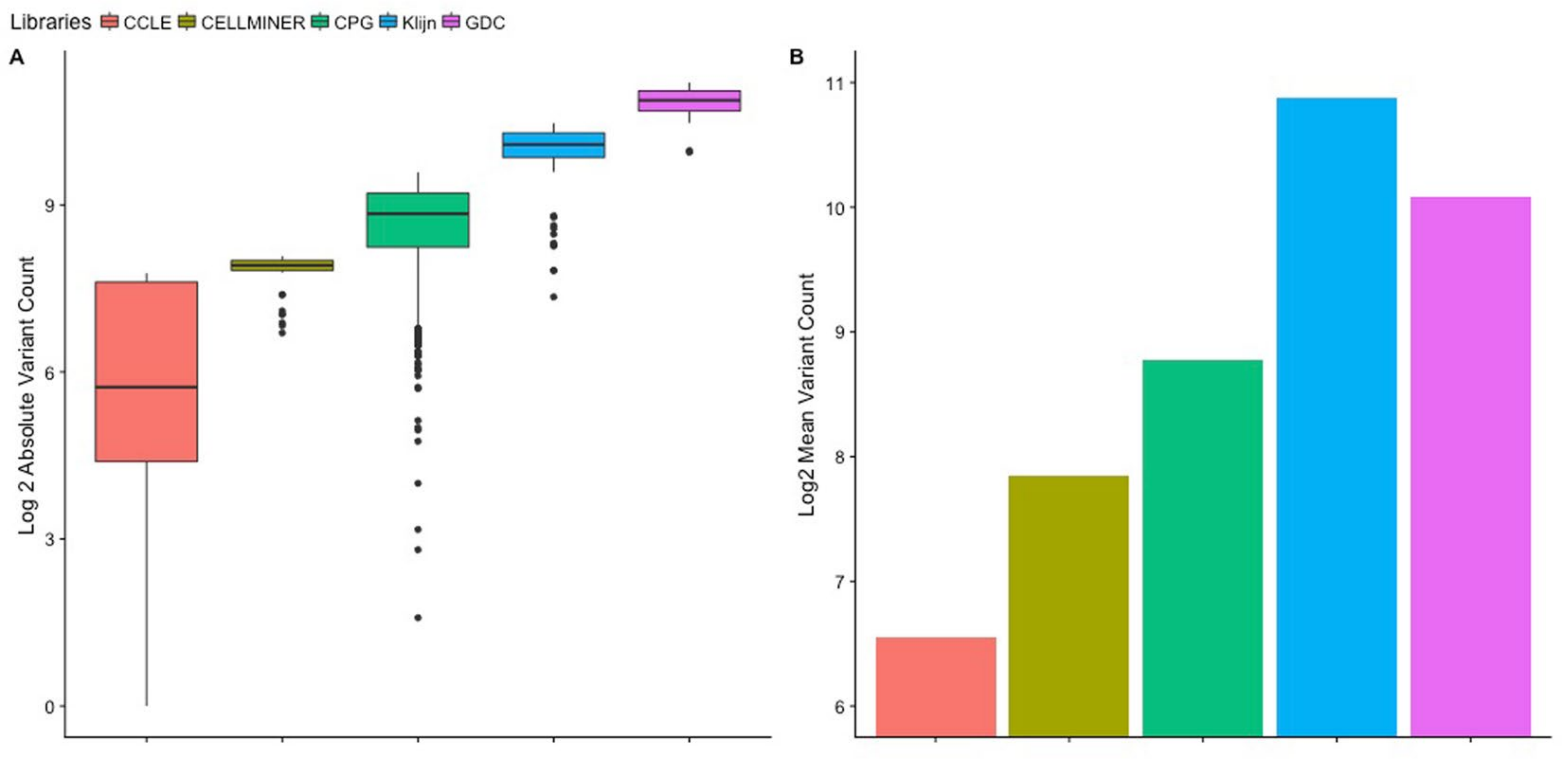
Heterogeneity of the benchmark data with respect to variant-counts. (**A**) Absolute amount of variants per benchmarked library. (**B**) Mean amount of variants per profile per benchmarked library. All repositories differed by at least one power of two with respect to the amount of variants they contain i.e. are heterogeneous. Whiskers depict the standard deviation of the mean variant-counts.

We benchmarked Uniquorn 2 on NGS data from 1612 RNA, 1080 DNA-exome and 904 targeted hybrid-capture sequenced CCLs from five repositories, in the following called libraries, which utilized four different sequencing technologies to adequately reflect the heterogeneity of a real-world scenario (Table 1 and Fig. 2). Four identification scenarios were benchmarked of which three were novel and not covered by Uniquorn 1: RNA-seq identification (Table 2), mixed RNA-seq and DNA-seq identification (Table 3), panel-seq identification (Table 4) and Uniquorn 1’s DNA-seq only scenario (Supplementary Material Table 1). It was benchmarked whether a CCL was correctly identified when comparing it to all reference CCL-profiles from all five reference libraries, leading to ~13 million CCL benchmark comparisons overall. Since a true positive prediction was only possible for about 11.000 of the ~13 million comparisons, our evaluations put special emphasis on the positive-predictive value (PPV).

**Table 2:** RNA-seq benchmark, showing the performance of Uniquorn 2 to identify full-transcriptome sequenced CCL-profiles. 1612 of such profiles were identified within five reference libraries containing 3596 DNA and RNA-seq sequenced CCLs. Columns 2 to 5 show key measures dependent on the mutational inclusion weight (see methods). Inclusion weights 1.0, 0.5 and 0.25 showed comparable performance with sensitivities above 95%. 0.5 is the default parameter setting of the Uniquorn 2 R-package.

Weight Threshold	1.0	0.5	0.25	0.0
Possible TPs	5309			
True positives	5096	5082	5071	4192
False negatives	213	227	237	1117
False positives	850	860	865	1411
Sensitivity %	96.0	95.7	95.5	79.0
Specificity %	99			
F1%	90.6	90.3	90.2	76.8
PPV %	85.7	85.5	85.4	74.8

**Table 3:** Real-world use-case benchmark. Uniquorn 2’s ability to identify CCL-profiles created and identified by RNA-seq, DNA-exome and DNA-hybrid-capture CCL-profiles is shown to determine the expected real-word use-case performance. 3596 CCLs that were sequenced and processed with various technologies and algorithms were identified (see Tables 1 and 2 for technologies). The sensitivity was comparable to the RNA-seq benchmark (Table 2) with the exception of inclusion weight 0.5 which resulted in a higher F1-score and PPV than weight 1.0. A performance drop can be observed for weight threshold 0.0 where all variants, informative and non-informative, were utilized.

Threshold	1.0	0.5	0.25	0.0
Possible TP	11512			
TP	10951	10945	10937	9843
FN	561	567	575	1326
FP	1128	1106	1139	4626
Sensitivity %	95.1	95.1	95.0	85.5
Specificity %	99			
F1%	92.8	92.9	92.7	85.5
PPV %	90.7	90.8	90.6	85.4

**Table 4:** Benchmark results for simulated panel-sized CCL-profiles. Uniquorn 2 achieves sensitivities of ~83%, ~82% and ~65% while constantly showing a specificity of higher than 99% at default parameters for panel-seq identification.

Panel	ClearSight	TruSight	Hotspot v2
Genes	151	94	49
Possible TP	11512		
TP	9505	9423	7525
FN	2007	2089	3987
FP	4591	4424	6097
Sensitivity %	82.6	81.9	65.4
Specificity %	99		
F1%	74.2	74.3	59.9
PPV %	67.4	68.1	55.2

### Cross-validation benchmark

The first finding was that Uniquorn 2 could effectively identify full-transcriptome sequenced CCL-profiles: with default parameters (Weight Threshold 0.5), Uniquorn 2’s sensitivity to identify RNA-sequenced CCLs reached 95.7%, its PPV 85.5% (Table 2). The rationale for choosing 0.5 as default weight threshold is shown in Supplementary Material (SM) Figs 1 and 2.

The second finding was that Uniquorn 2 could effectively identify CCL profiles in a real-word scenario: Heterogeneously created RNA-seq and DNA-seq CCL-profiles had to be identified by equally heterogeneously created reference CCL-profiles what resulted in an average sensitivity of 95% and average PPV of 90% (Table 3). Both RNA-seq and mixed-seq benchmarks showed extremely high specificity values (99.9% and higher) which were caused by the very large number of true negative predictions.

The 3596 available reference CCL profiles were reduced to the genomic regions covered by three of the most widely utilized ClearSight, TurSight and Hotspot v2 panels to simulate panel-seq benchmark profiles. Identification of the resulting 3 * 3596 = 10788 panel-profiles revealed as third finding that panel-seq profiles could be successfully identified with an average sensitivity of 82% and PPV of 68% if the panel covered more than 100 genes (Table 4). Panels covering less than 100 genes were significantly less suited for CCL-identification with an average sensitivity of 60% and a PPV of 55%. Specificity always remained higher than 99%. False-negative and false-positive identifications were found to be predominantly caused by CCLs-profiles that covered less than 100 genes.

**Figure 3:**
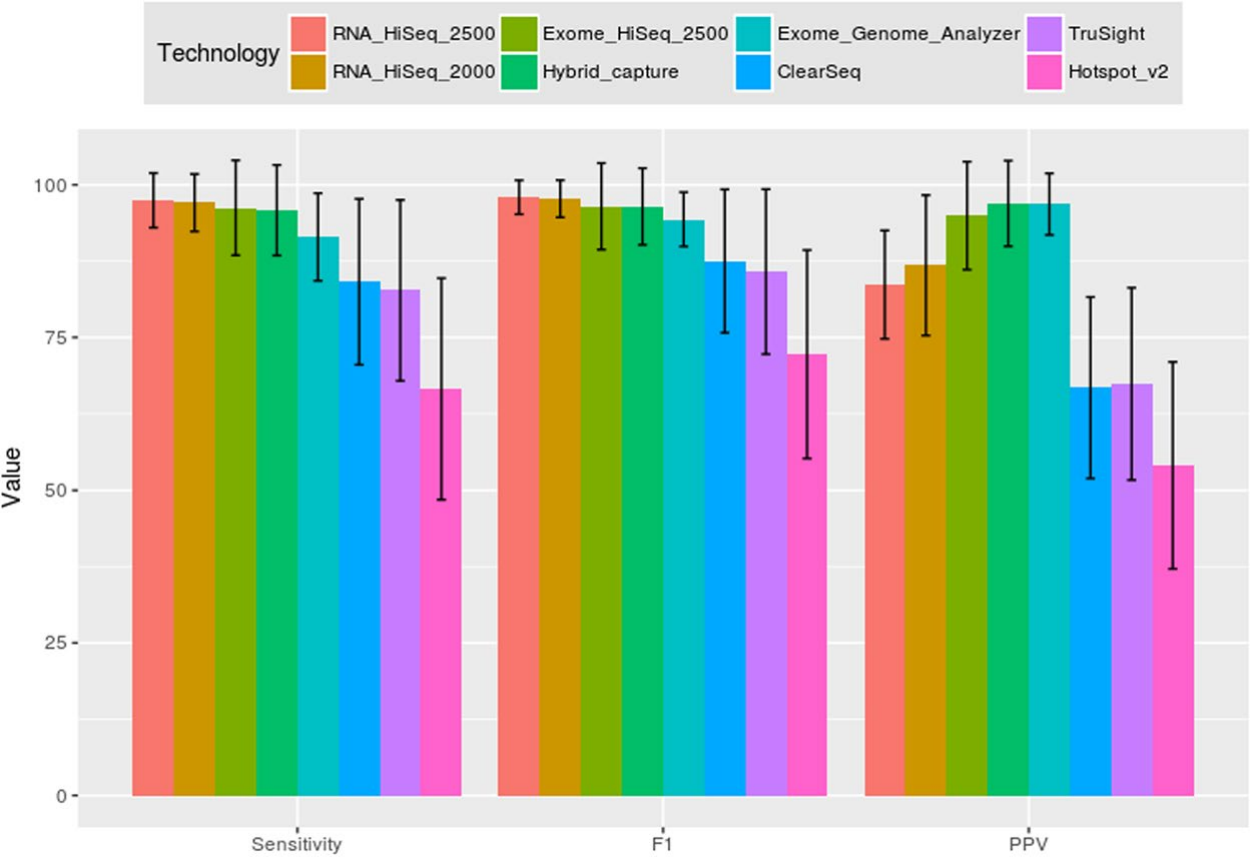
Relationship between data heterogeneity and identification performance. CCL profile sequenced and processed by vastly different technologies and algorithms were identified and determined whether Uniquorn 2’s identification performance remained robust in spite of the data heterogeneity. Bars depict average performance, whiskers standard deviation. Profile sizes of the query CCL shrink dramatically from left (~2**10 variants) to right (~50 variants). Sensitivity and F1 score are highest when full transcriptome profiles are used and lowest for small panel-seq profiles but remain robust when faced with different technologies. In general, PPV decreases with the profile size with the exception of exome-seq and hybrid-capture technologies, which show a higher sensitivity than the more sizable full-transcriptome technologies.

Subsequently, it was analyzed what factors caused Uniquorn 2 to incorrectly classify i.e. identify a CCL-profile and it was determined that technological heterogeneity does not significantly impact Uniquorn 2’s sensitivity and F1 score (Fig. 3). However, although sensitivity and F1 score remained robust with respect to the utilized technology, sensitivity showed a strong positive correlation (r of 0.7) with the amount of genes covered by a profile. The uncovered sensitivity to amount-of-covered-genes relationship is depicted in SM Fig. 3 and the benchmark results for each library are shown in SM Fig. 4. The PPVs showed a limited bias with respect to utilized sequencing technology and no log-linear relationship to the amount of covered genes.

## Discussion

Uniquorn 2 is optimized for the identification of CCLs whose variant profiles were obtained by heterogeneous technologies and diverging computational processing pipelines. Thus, it complements established methods by addressing some of their key limitations: 1) The physical CCL sample is not required, as it is, for instance, in the case of STRs-based identification, 2) Uniquorn 2 is agnostic to sequencing technology and thus able to reuse data provided by the creators of CCL libraries. We benchmarked the performance of the algorithm in high-diversity scenarios, which we consider best mimic the real situation, in laboratories dealing with CCL, confirming its ability to cope with various sequencing and data-processing technologies (Table 1). This considerably extends the functionality of Uniquorn 1 to also handle RNA and panel-sequenced CCLs (Tables 2 and 4).

Panel-seq profiles were simulated by reducing the amounts of covered genes of the 3596 available profiles from about 22000 down to 151, 94 and 49 covered genes, respectively. Differences in the identification efficiency of the benchmarked panels (Agilent ClearSight, Illumina TruSight, Thermo-Fisher Hotspot v2) was therefore caused by differing amounts of covered genes and not due to heterogeneous technology since the variants call within the covered genes were identical for each panel. Significant differences regarding sensitivity, F1-score and PPV were detected between the panels, indicating that not the sequencing technology (Fig. 3) but the number of covered genes is most influential with respect to how efficiently a CCL profile can be identified (SM Fig. 3). Remarkably, the identification efficiency of panel-seq profiles was merely 12% to 13% lower than the efficiency measured for full transcriptome sized CCL-profiles although the panels covered orders of magnitude less genes than the full-transcriptome profiles. An exception was the hotspot v2 panel which showed a significantly decreased sensitivity of 65% which was 30% lower than the full-transcriptome profile identification but as well only covered 49 genes.

By manual inspection of benchmark results (SM Table 1) we found that false-negative identification is associated with CCLs that had diverged significantly from their origin due to long-term subclonation or exposure to drug treatment e.g. the CEM-2, Jurkat and CCRF-CEM CCLs. This finding is supported by reports of the same phenomenon for the same CCLs when STR-identification was applied^15^. False-negative predictions where furthermore frequently associated with CCLs whose relationship-status could not be fully resolved due to an unclear nomenclature: E.g. when it was unclear whether CCLs with a similar name were different or identical CCLs or in the case of false-positive, whether CCLs with different names were nevertheless identical but counted as false-positive by the gold-standard (SM Table 2). We summarized numerous labeling inconsistencies (SM Table 3). Thus, low variant-counts and an unclear relationship caused by the absence of a generally applied CCL-nomenclature system are still the dominant causes of incorrect predictions.

Uniquorn 2 complements established methods in particular when those cannot be applied e.g. due to absence of a physical sample. The Uniquorn 2 method supports quality-assurance procedures in high-CCL-throughput laboratories since it seamlessly integrates into analysis pipelines to serve as a quick test for in-house or procured third-party CCL-profiles. The Uniquorn 2 method is freely available as Bioconductor R-package ‘Uniquorn’ (contains both Uniqorn 1 & 2) and can be easily implemented.

Users of Uniquorn 2 can utilize their own sets of CCL-profiles as reference. However, as the run time of Uniquorn 2 is very low, it is advisable to always include a wide range of reference profiles to also detect unexpected contamination. The CGP and CCLE repositories contain 1695 CCL-profiles while showing a low false-negative rate as references and are freely available. The ‘Uniquorn’ R-package is ported with the limited NCI-60 reference panel but a tutorial that enables researcher to easily utilize the 1695 CGP and CCLE CCLs is documented in the ‘Uniquorn’ Bioconductor vignette, see SM Table 1. The Klijn *et al*. and GDC CCL-repositories show suitable identification characteristics and can be obtained by application at the European Genome Archive.

Detailed analyses of factors influencing the identification of CCL-profiles such as SNP filtering are indicated to further improve the Uniquorn 2 method. Moreover, a further extension to non-cancer CCLs, single or methyl-sequenced CCLs are viable subjects for future work to further expand the range of research fields which can utilize the Uniquorn 2 method.

## Material and Methods

### General concept

We define the profile of a given sample *c* as the set of its variants *var(c)* - small INDELs and SNVs - that were obtained from genotyping *c* by some form of (next-generation) sequencing, where a variant is characterized by its start position, end position, and chromosomal location. Two variants are considered identical when all these values are identical. Given a sample *q* (query) whose identity is to be confirmed and a reference CCL-library *L*, Uniquorn 2 tests whether *q* was derived from any of the CCLs from *L* by comparing the *var(c)* profile of *q* to the profiles of all CCLs from *L*. For simplicity, we use from now on *q* to denote the profile of the query sample and *l* to denote the profile of a CCL from *L*. Note that Uniquorn 2 can also be used for searching *q* in multiple reference libraries. We assume that a single library consists of homogeneous CCL-profiles with respect to their laboratory of origin, technology, and bioinformatics processing, however we also assume that the technology used to obtain *q* is not the same as in any of the libraries; searching across technologies is one of the core abilities of our algorithm. We assume that all libraries are independent of one another.

**Figure 4:**
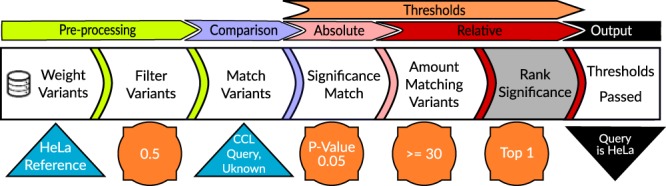
Uniquorn 2 workflow. Uniquorn 2 selects small variants strongly associated with one or few reference CCL-profiles and matches these to the variants of the query profile. When the resulting overlap of characteristic variants between a reference and query passes all significance thresholds, the query is identified as the reference CCL. The gray-shaded area indicates the distinction of the Uniquorn 1 identification method which did not contain the second relative threshold.

Uniquorn 2 classifies *q* as identical to one or multiple *l* *∈* *L* by rejecting the null hypothesis *h*_0_ which states that the profiles of *q* and *l* overlap due to chance. An overview of the workflow is shown in Fig. 4. In the following, we describe the algorithm in detail.

### Detailed workflow

1. Characteristic variants
  For comparing profiles, Uniquorn 2 considers only the variants that are characteristic for *l* in *L*. We find these variants by computing a weight *w(v)* for every variant *v* found in any of the CCLs of *L* as follows:
  $$w(v)\,:\,={2}^{-f(v)-1}$$
  where *f(v)* is the frequency of *v* in *L*. We consider *v* characteristic if *w(v)* is smaller than a user-selected threshold (default 0.5; the determination of default parameters: SM Figs 1 and 2).
2. Confidence score calculation
  Uniquorn 2 compares the profile of *q* to all profiles in *L*. For a given *l*, let *m*_*l*_ be the size of the profile overlap of *q* and *l*, and let *m*_*L*_ be the number of variants contained in *q* and any CCL from *L*. We first compute the probability *P*_*l*_ = *P(m*_*l*_ *| q*, *l*, *L)* to observe an overlap of size *m*_*l*_ between the profiles of *q* and *l* by chance. Computing *P*_*l*_ requires the probability *p*_*l*_ of finding a single match between *q* and specific *l*, which we estimate as the relative size of the profile of *l* in *L*:
  $${p}_{l}\,:\,=\frac{|l|}{|L|}$$
  This formula mitigates the fact that profiles with a high variant-count are more likely to be matched by chance than CCL-profiles with a lower variant-count. Using *p*_*l*_, we calculate *P*_*l*_ as 1 minus the binomial cumulative distribution function based on the formula:
  $${P}_{L}\,:\,=1-{\sum }^{}(\begin{array}{c}{m}_{L}\\ i\end{array}){({p}_{l})}^{i}{(1-{p}_{l})}^{{m}_{L}-i}$$
  Subtraction from 1 was chosen following Mi *et al*.^19^ to obtain p-value suitable probabilities. We finally define the confidence *CS*_*q*,*l*_ of *q* being derived from the same CCL as:
  $${{\rm{CS}}}_{{\rm{q}}{\rm{,}}{\rm{l}}}\,:\,=-\,1\cdot {\mathrm{log}}_{e}({P}_{L})$$
3. Quantification of spuriousness and filtering of false positives
  A particular problem when comparing profiles obtained from different genomic areas, such as a whole transcriptome derived profile with a panel-derived profile, is their strongly diverging count of variants (Fig. 2). For instance, a CCL library might have been characterized by RNA-seq, leading to significant amounts of ~2^9^–2^12^ many variant-calls per CCL-profile, whereas the query might have been subjected to panel-seq, which yields much smaller profiles (for a panel of 100 genes, typically not more than few hundred variants are called). In such cases, there is an increased chance of observing spurious matches; however, these often cluster, i.e., we find the same (false) match in multiple CCLs of the library being studied. Furthermore, false positive predictions show an amount of matching variants that is comparable to the average amount of matching variants in *L*. To filter such cases, we first quantify the size-induced spuriousness *SP*_*L*_ of the matches between *q* and all *l*. *SP*_*L*_ is computed as the integral of the beta function based on the ratio between the average amount *m*_*avg*_ and the maximum amount *m*_*max*_ of matching variants in *L*. We chose the integral of the beta-function due to the integral’s skewness, two-parameter positive integer domain for *m*_*avg*_, *m*_*max*_ and real-valued co-domain between and including 0 and 1.
  $${{\rm{SP}}}_{L}\,:\,=\frac{\Gamma ({m}_{{\rm{\max }}})\cdot \Gamma ({m}_{{\rm{avg}}})}{\Gamma ({m}_{{\rm{\max }}}+{{\rm{m}}}_{{\rm{avg}}})}$$
  In a second step, we filter all overlaps with less matches than threshold *T*_*L*_ to exclusively retain overlaps that show a higher number of matches than expected by chance:
  $${T}_{L}\,:\,=(\frac{{m}_{{\rm{avg}}}+{{\rm{m}}}_{{\rm{\max }}}\cdot {{\rm{SP}}}_{L}}{1-{{\rm{SP}}}_{L}})$$
4. Rejecting the null hypothesis

Eventually, three conditions have to be fulfilled for rejection of *h*_0_:

A) *CS*_*q*,*l*_ has to be equal or greater than threshold *t* (default value is *t* = 3)

B) *CQ*_*q*,*l*_ must rank among the top-*k* positions of all *l* in *L* (default value *k* = 2)

C) *m*_*l*_ must be significantly greater than the average overlap of all *l* in *L*, *TL*.

### Evaluation

We benchmarked Uniquorn 2 using 3596 CCL-profiles derived from 1516 distinct CCL-samples from five libraries, each characterized by a different technology (Table 5). We utilized the 3596 profiles both as reference and as queries, resulting in 3596 identification tasks and roughly 13 Million individual comparisons. Each query profile possessed between one and nine matching reference profiles (median = 3) because many CCLs are contained in more than one library. In addition to obtaining key performance indicators (Tables 2–4), we also assessed whether the performance was biased related to certain properties of the profiles such as sequencing technology (Fig. 3 and SM Fig. 4).

**Table 5:** Heterogeneity of CCL-profiles utilized for the benchmarks.

Technology	Source	GenotypedGenes	Variant calling software	SNP Filtering*
RNA-seq	Klijn *et al*.	Expressed alleles only	GATK RNA	None
	GDC		FreeBayes	
Hybrid-capture	CCLE	1651	MuTect^24^	>0.01
			Pindel^25^ Caveman^26^	
Exome-seq	CGP	20965		>0.0
	CellMiner	>20k	GATK DNA^27^	None

Data differs with respect to sequencing technology, variant calling algorithms, SNP-filtering, and number of covered genes. Variants within GDC and Klijn *et al*. repositories were manually called by first utilizing the Trimmomatic^28^ and the STAR^29^ aligner and a subsequent diverging variant calling step: the GATK-RNA variant caller^30^ was utilized for data from Klijn *et al*. and the FreeBayes^31^ variant-caller for GDC data to increase the heterogeneity of the benchmarked data. *SNPs were pre-filtered by the creators of the data based the SNPs’ minor allele frequency.

Sensitivity was defined as the fraction of all predictions which correctly predicted that two CCLs profiles were similar and specificity as the fraction of all predictions which correctly stated that two CCL profiles were not similar.

### Gold-standard creation

We created a gold-standard based on CCL names and literature research. Firstly, names of CCLs were either parsed from the VCF-files directly (Cellminer, GDC, Klijn *et al*.) or extracted from the meta-file that aggregated the variant-calls of all CCL-profiles into a single document (CCLE, CGP). Secondly, a pre-processing step removed all non-alpha-decimal characters and spaces from the names and capitalized the processed names. CCLs that differed only by a prefix or by a suffix, such as *MDA-MB-435* and *MDA-MB-435S*, were considered candidates for being identical and validated using literature. Also, collisions of different CCLs that had the same name after the pre-processing e.g. *TT* and *T*.*T* were resolved by literature research. This process resulted in 11508 identity-relationships of which 5309 are based on RNA-seq profiles. SM file 2 contains the gold-standard, SM File 3 contains the identity-definitions based on reports and a link to the reports where needed.

### Panel data creation

The CCL profiles of all libraries we considered were obtained by either DNA or RNA sequencing. However, labs often only perform panel sequencing with their samples to save on cost and labor^20^. To test the capability of Uniquorn 2 to identify a panel-sequenced sample within an RNA or DNA sequenced library, we created synthetic panel-seq profiles by removing all variants from a profile that fall outside the region of three predefined panels, i.e., gene set. Firstly, we formatted all profiles into the VCF-format and secondly bedtools^21^ intersected all VCF-files with BED-files containing the genomic coordinates of the panels. The TruSight’s BED-file (trusight_cancer_manifest_a.bed) was obtained from www.illumina.com. The websites of the Hotspot v2 (www.thermofisher.com) and the ClearSeq panel (www.agilent.com) did not provide the panels’ genomic-coordinates in BED but comma-separated format and thus we manually converted the comma-separated files into the BED-format using BioMart^22^.

### Data acquisition

We procured the data either in the VCF-format or as BAM-files (Table 6). BAM-files were deconvolved into FASTQ-files and conscientiously processed with different variant calling algorithms to obtain VCF-files (Table 2). The CCL-profiles from the CGP and CCLE repositories were extracted from the meta-files and transformed into VCF-files. R version 3.5.1 (2018-07-02) was utilized on a Linux Debian Mint operating system and benchmarks performed with the Bioconductor ‘Uniquorn’ package 2.0.0^23^.

**Table 6:** Acquisition of the benchmark data. Origin and name of utilized files used for the benchmark are shown. Klijn *et al*.^32^, GDC^2^ CGP^33^, CCLE^2^ and Cellminer^3^ were procured.

Library	URL	File(s)	Date
Klijn *et al*.	Ebi.ac.zk	BAMs	July 16^th^ 2017
GDC	Portal.gdc.cancer.gov	BAMs	May 24^th^ 2017
CGP	Sftp-cancer.sanger.ac.uk	CosmicCLP_MutantExport.tsv	January 13^th^ 2016
CCLE	Broadinstitute.org/ccle	CCLE_hybrid_capture1650_hg19_NoCommonSNPs_CDS_2012.05.07.maf	
Cellminer	Discover.nci.nih.gov/cellminer	VCFs	

## Electronic supplementary material

Supplementary Figures

SM_Dataset_1_Benchmarks.xlsx

SM_Dataset_2_Goldstandard.xlsx

SM_Dataset_3_Known_relationships.xlsx

## Data Availability

Exclusively publicly available data has been utilized for benchmark purposes, see methods section for further information.
