# Supplementary material for: Robust in-silico identification of Cancer Cell Lines based on RNA and targeted DNA sequencing data
Source: Sci Rep. 2019 Jan 23;9:367. doi: 10.1038/s41598-018-36300-8 (PMC6344579; doi:10.1038/s41598-018-36300-8)
Supplement: Supplementary file 1 — Supplementary Figures [file 41598_2018_36300_MOESM1_ESM.pdf]

Robust in-silico identification of Cancer Cell Lines based on RNA and targeted DNA sequencing data

Raik Otto[1],*, Jan-Niklas Rössler[1], Christine Sers[2,3], Soulafa Mamlouk[2,3], Ulf Leser[1]

1 Knowledge Management in Bioinformatics, Institute for Computer Science, Humboldt-Universität zu Berlin, Unter den Linden 6, 10099, Berlin, Germany

2 Charité Universitätsmedizin Berlin, Institute of Pathology, 10117, Berlin, Germany

3 DKTK, German Consortium for Translational Cancer Research, Partner Site Berlin

* To whom correspondence should be addressed. Tel: 0049 030 2093 3086; Fax: 0049 030 2093 5484; Email: raik.otto@hu-berlin.de

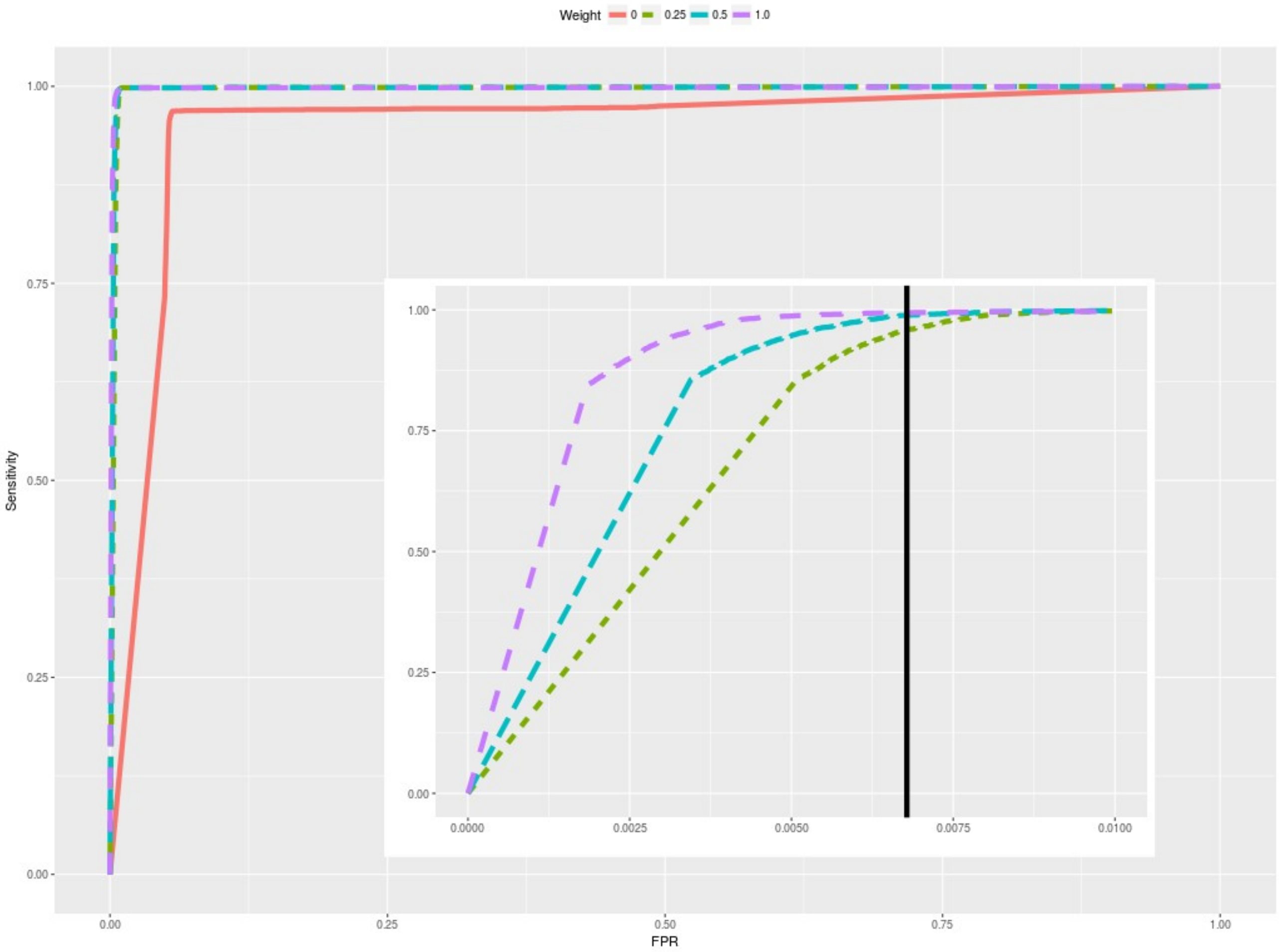

SM Figure 1: Determination of the default confidence score for DNA and RNA-seq identifications based on the cross-identification benchmark's Receiver-Operator-Curves (ROC). The ROC-curve iterates over the confidence score compares a score's associated sensitivity and associated specificity. It can thus be seen how the overall default weight threshold was chosen as the optimal ratio between sensitivity and specificity. The embedded plot shows the same ROC plot with an adjusted FPR-axis range to visualize the ROC curve of inclusion weight 0.0. The vertical black line shows the Uniquorn default threshold sensitivity to specificity ratio. The identified thresholds were set as default within the 'Uniquorn' package.

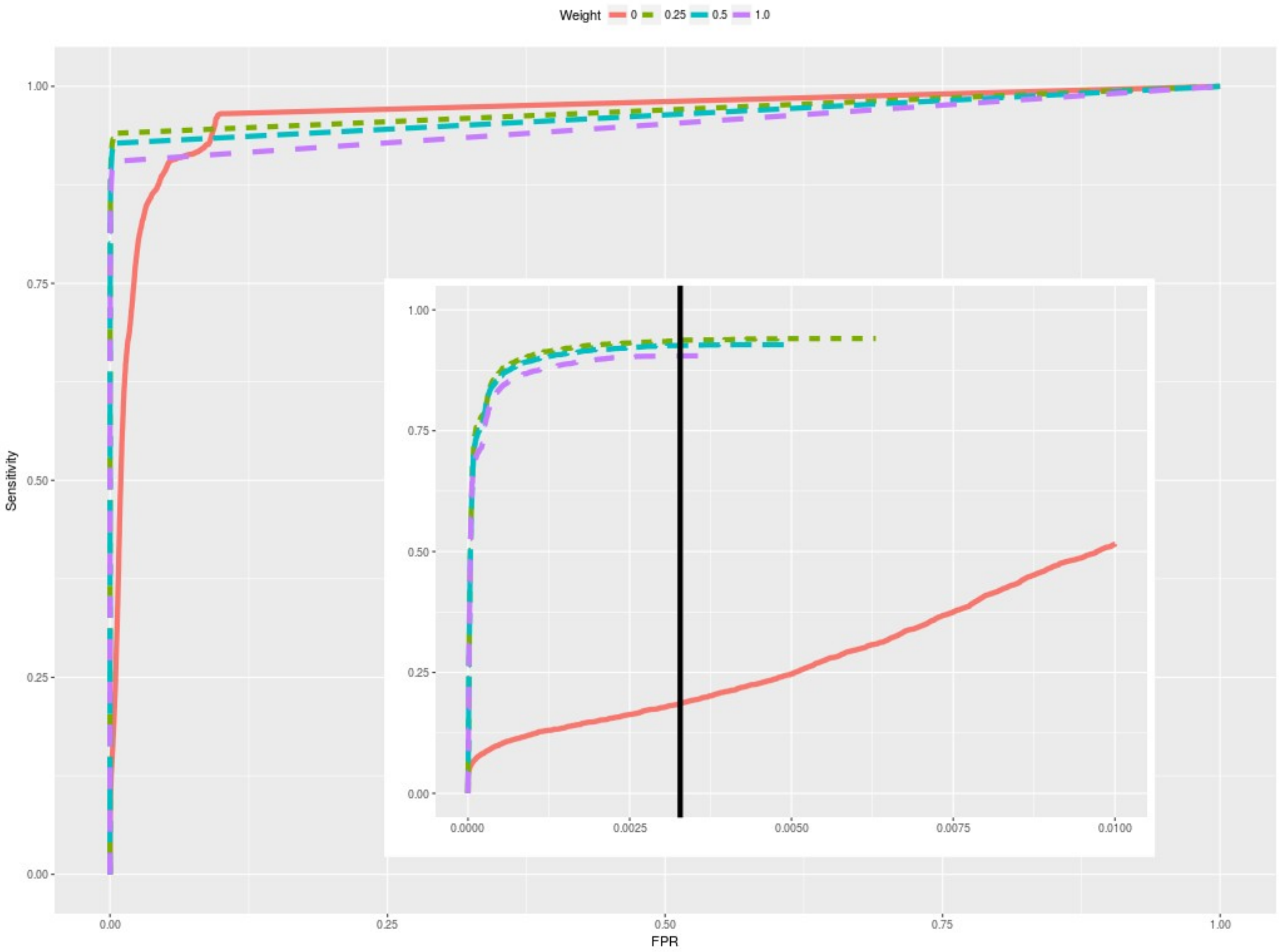

SM Figure 2: Determination of the default confidence score for panel-sequenced CCLs based on the cross-identification benchmark's Receiver-Operator-Curves (ROC) of the TruSight Cancer Panel. The Figure description is identical to Figure 2. Inclusion weights 0.5 and 0.25 show the best ratio between sensitivity and False Positive Rate. The identification of different optimal threshold for panel and non-panel-sequencing indicates, that users should actively adapt the identification threshold.

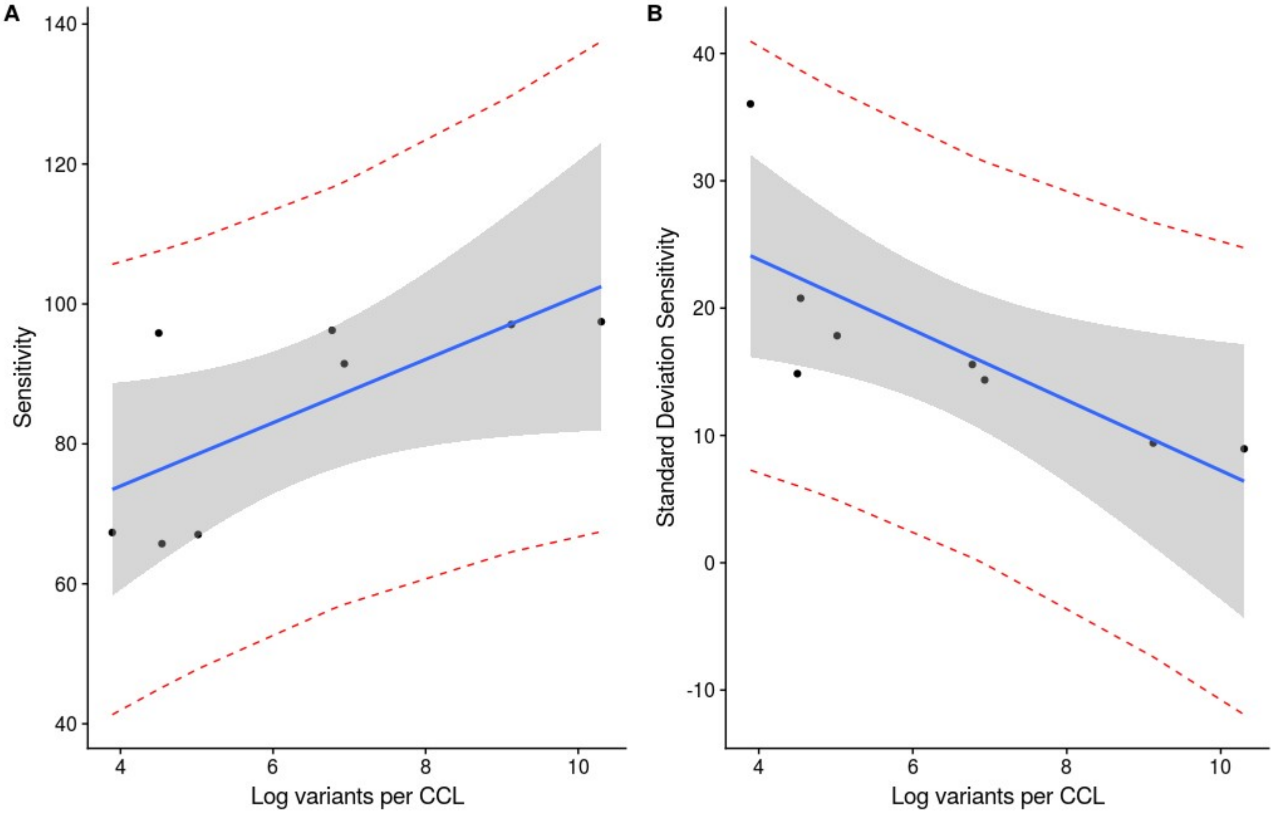

SM Figure 3: Correlation between sensitivity and average variant-count per CCL within a library. A linear regression depicts the relationship between the variant count of a CCL-profile and the sensitivity with which CCL-profiles can be identified. A log-linear correlation between a library’s average number of variants per contained CCL-profile and the sensitivity with which these profiles exists. Sensitivity is correlated with an r of 0.7 and regression p-value of 0.041 and standard deviation with an r of -0.75 and p-value of 0.03. Shaded areas indicate the regression standard error and dashed lines indicate the regression’s 95% confidence interval.

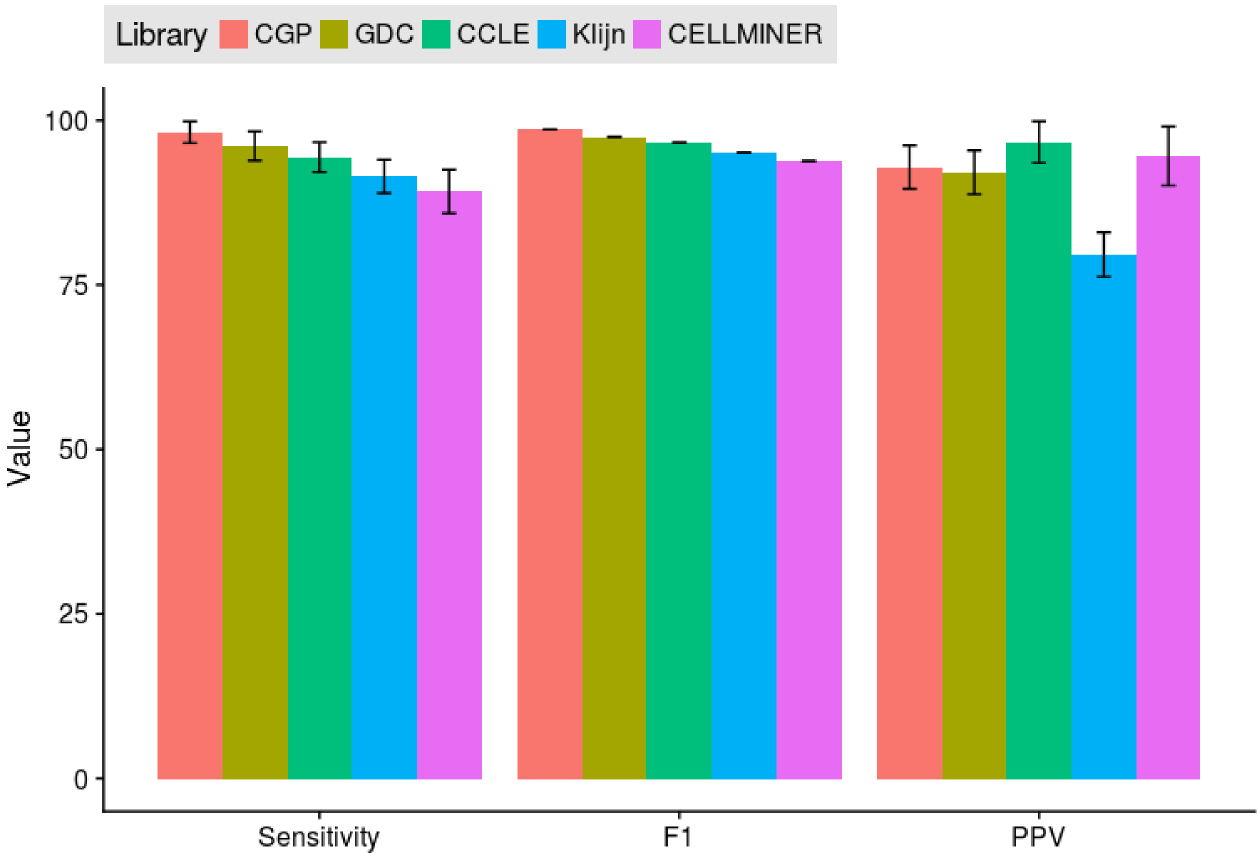

SM Figure 4: Benchmark results split by library. Sensitivity and F1 value do not show a significant change between libraries. Overall, the benchmark results remain robust with the exception of the PPV for the Klijn library which is a minor outlier due to slightly worse PPV.
